# The fecal bacterial microbiota of bats; Slovenia

**DOI:** 10.1371/journal.pone.0196728

**Published:** 2018-05-23

**Authors:** Modest Vengust, Tea Knapic, J. Scott Weese

**Affiliations:** 1 Veterinary Faculty, University of Ljubljana, Ljubljana, Slovenia; 2 Slovenian Museum of Natural History, Ljubljana, Slovenija; 3 Dept of Pathobiology, Ontario Veterinary College, University of Guelph, Guelph, Ontario, Canada; Free University of Bozen/Bolzano, ITALY

## Abstract

**Methods:**

Fecal samples were collected from 92 bats in Slovenia, consisting of 12 different species, and the bacterial microbiota was assessed via next generation sequencing of the 16S rRNA gene V4 region.

**Results:**

Sequences were assigned to 28 different phyla, but only Proteobacteria, Firmicutes, Bacteroidetes and Actinobacteria accounted for ≥1% of sequences. One phylum (Proteobacteria), one class (Gammaproteobacteria), three orders (Pseudomonadales, Lactobacillales, Bacillales), four families (Enterobacteriaceae, Pseudomonadaceae, Staphylococcaceae, Carnobacteriaceae), and five genera (*Pseudomonas*, *Staphylococcus*, *Carnobacterium*, an unclassified Enterobacteriaceae, *Acinetobacter*) accounted for 50% of sequences. There were no significant differences in the relative abundances of any phyla between bat species, but various differences were noted at lower taxonomic levels, such as Enterobacteriaceae (*P* = 0.007, most abundant in *M*. *blythii*), Pseudomonadaceae (*P* = 0.007, most abundant in *Rhinolophus hipposideros*) and Chlamydiaceae (*P* = 0.04, most abundant in *Myotis myotis*). There were significant differences in richness between species in both adults and juveniles/subadults, but there was no impact of sex on any alpha diversity index. When only adults are considered, there were significant differences in community membership between *M*. *blythii* and *M*. *emarginatus* (*P* = 0.011), and *M*. *blythii* and *R*. *hipposideros* (*P* = 0.004). There were also significant differences in community structure between *M*. *blythii* and *M*. *emarginatus* (*P* = 0.025), and *M*. *blythii* and *R*. *hipposideros* (*P* = 0.026). When adults of the four main species were compared, 14 OTUs were identified as differentially abundant using LEfSe. Only one difference was identified when comparing *R*. *hipposideros* adults and juvenile/subadults, with *Klebsiella* over-represented in the younger bats.

**Conclusions:**

Bats have a complex and diverse microbiota with a high relative abundance of Proteobacteria. The relevance of this difference is unclear and requires further study. Differences in the microbiota were observed between bat species, perhaps reflecting different diets and environmental exposures.

## Background

Bats (order Chiroptera) are a diverse group of mammals adapted to a variety of ecological niches across the globe. They are the only mammals capable of true flight, which is essential for their biology. They are able to migrate over long distances, creating opportunities for diverse exposure and widespread dissemination of microbes. Bats that reside in temperate zones such as northern and central Europe migrate south to warmer geographical locations or hibernate to avoid cold environmental temperatures. [1]

Focus on bats often pertains to their status as reservoir host for several emerging zoonotic viral pathogens, as well as many common human and animal viruses. [2–4] They have developed a benign phylogenetic relationship with several viral and non-viral intracellular pathogens, with some of them having or potentially having a zoonotic character. [2] Their presence in a variety of human habitats, and their ability to migrate over larger geographical distances make them significant sources of transmission of pathogens to humans, livestock and wildlife species. [5, 6] Migration habits and their tendency to share roosting sites with other migrating and non-migrating bat species also enables horizontal spread of pathogens within and among bat species. [2, 5] Such behaviour is also critical for transmission of bat specific viral and non-viral infectious diseases, [7, 8] including those with a potential negative impact on the sustainability of bat population, [7, 9] as evident from a significant decline in North American bat population due to White-nose syndrome caused by *Pseudogymnoascus destructans*. [10, 11]

Attention is increasingly being paid to the importance of the broad microbiota communities, or microbiotas, present in or on animals. These microbiotas play important but poorly defined roles in health and disease. Through diverse effects such as pathogen inhibition, co-aggregation and regulation of the immune system and metabolism, microbial communities can have profound effects on their host and on other microorganisms (e.g. viruses, fungi, protozoa). An important aspect of determining the role of microbiotas in the prevention or pathogenesis of disease is understanding what constitutes a normal microbiota and factors that can influence such microbial communities. The objective of this study was to describe the fecal microbiota of bats collected during their autumn migration across central Europe.

## Materials and methods

### Study population and sampling

**Fig 1:**
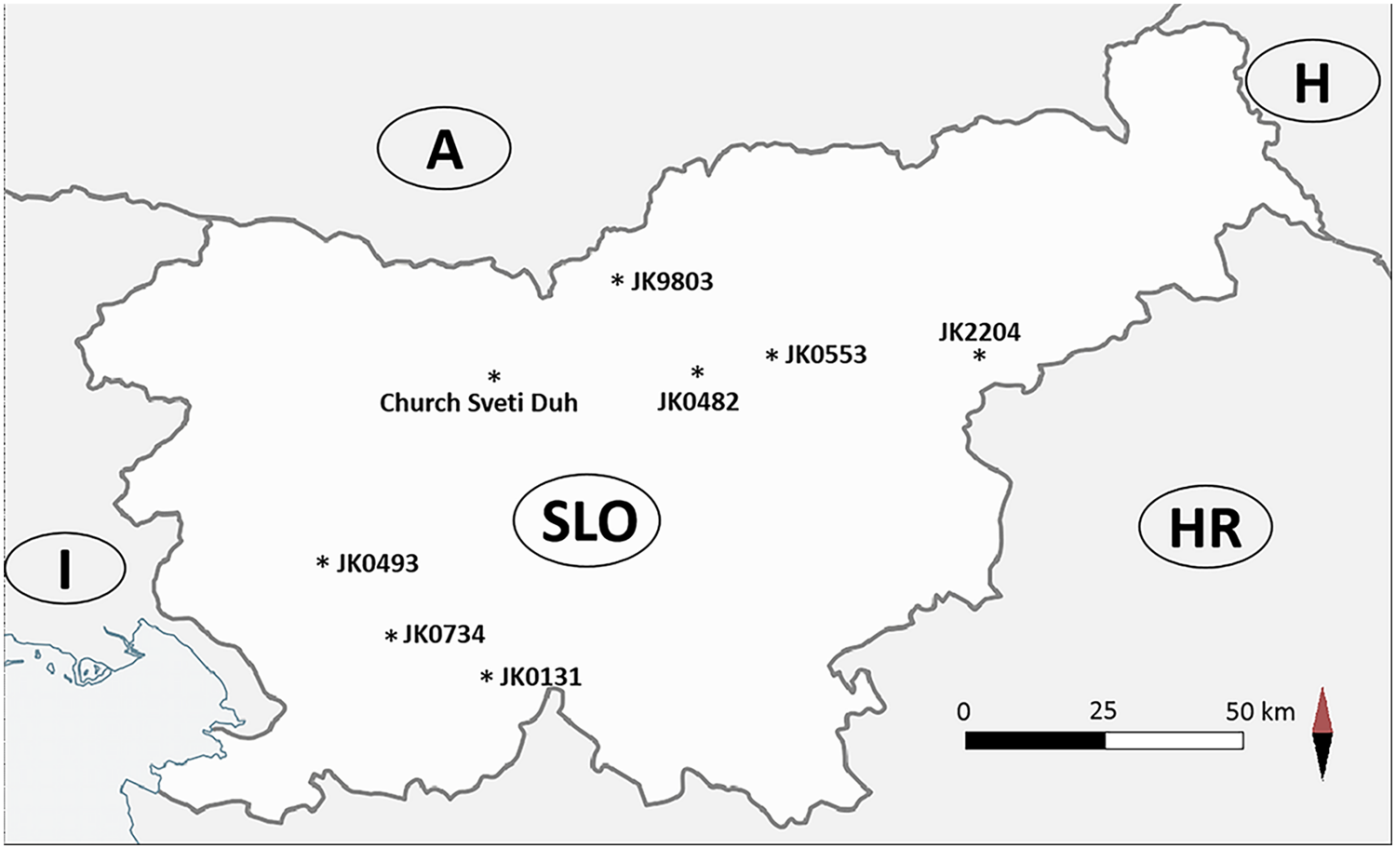
Cave sampling sites across Slovenia (SLO). Depiction of bat sampling sites in Slovenia.

Sampling of bats was conducted in 8 different parts of Slovenia (Fig 1, Table 1) during their autumn migration across central Europe from August to September 2014. Bats were captured with mist nets and placed individually in clean custom made bags by one of the authors (TK) and her team from the Centre for Cartography of Fauna and Flora. [12] Bats were speciated based on their morphology, and age and sex were determined for each animal. [13] All bats were released within 30 min of their capture at the capture location. Fecal samples (guano) were collected from bats only if naturally excreted before they were placed into a bag. Feces was caught with a sterile glove (Ansell Ltd, UK), transferred into 2 mL sterile tubes (Eppendorf Tubes, Germany) and stored at -20°C within four hours of collection. If multiple fecal pellets were excreted they were pooled.

**Table 1:** Bat species collected from different caves in Slovenia.

Cave and species	No. of samples
Cave: Belojaca (JK2204)	8
*Barbastella barbastellus*	1
*Miniopterus schreibersii*	2
*Myotis bechsteinii*	1
*Myotis emarginatus*	1
*Myotis myotis*	1
*Myotis nattererii*	1
*Rhinolophus ferrumequinum*	1
Cave: Ciganska jama (JK0493)	4
*Barbastella barbastellus*	1
*Myotis bechsteinii*	1
*Myotis daubentonii*	1
*Rhinolophus hipposideros*	1
Cave: Golobina (JK0131)	3
*Myotis daubentonii*	2
*Myotis emarginatus*	1
Cave: Jama hudega bika (JK9803)	23
*Myotis emarginatus*	9
*Myotis nattererii*	1
*Rhinolophus hipposideros*	13
Cave: Pekel pri Zalogu (JK0553)	3
*Myotis myotis*	3
Cave: Predjamski sistem (JK0734)	17
*Barbastella barbastellus*	1
*Miniopterus schreibersii*	1
*Myotis blythii*	11
*Myotis daubentonii*	1
*Myotis myotis*	1
*Pipistrellus pipistrellus*	2
Cave: Skadovnica (JK0482)	30
*Barbastella barbastellus*	1
*Myotis bechsteinii*	1
*Myotis daubentonii*	1
*Myotis emarginatus*	8
*Myotis myotis*	2
*Myotis nattererii*	1
*Plecotus auritus*	1
*Rhinolophus hipposideros*	15
Steeple (Church Sveti Duh)	4
*Myotis myotis*	4
Sum	92

The study was carried out during the program “Monitoring of population of selected bats species in years, 2014/2015, [14] carried out by Centre for Cartography of Fauna and Flora with approval of Slovene Ministry of the Environment and Spatial Planning (document No.: 35601-35/2010-6). Bats were handled as specified in the European and Slovene Nature conservation regulations.

### DNA extraction and 16S rRNA gene PCR

A commercial kit (E.Z.N.A. Stool DNA Kit, Omega Bio-Tek Inc., Doraville, Georgia, USA) was used for DNA extraction and DNA quantity and quality were assessed by spectrophotometry (Nano Drop, Roche, Mississauga, Canada). The V4 region of the 16S rRNA gene was then amplified using primers 564F and 785R [15] and amplicons were sequenced by Illumina MiSeq using 2X250 chemistry, providing fully overlapping paired end reads. Negative (PCR water instead of template) controls were included in all runs.

### Data analysis

Analysis was performed using the open-sourced bioinformatics package MOTHUR (v1.35). [16] After assembly of paired end reads, sequences underwent a series of quality control filters to remove those that contained any ambiguous base calls, were inconsistent with the target amplicon size (240 bp), contained runs of holopolymers >8bp in length, or did not align with the correct 16S rRNA gene region. Uchime [17] was used to detect chimeras, which were subsequently removed. Taxonomy was assigned using the ribosomal database project 16S rRNA dataset [18]. Relative abundances of taxa were compared between the four main bat species by Wilcoxon test, with the Benjamini-Hochberg technique used to adjust *P* values false discovery rate. Analysis of less common species was not performed because of the low statistical power. Alpha diversity was calculated using Chao1 richness, inverse Simpson’s diversity and Shannon’s evenness tests, and compared using Steel-Dwass or Wilcoxon rank sum tests. Logistic regression was used to evaluate the impact of age, gender and species on alpha diversity indices. Chi-square test was used to compare the proportion of adult samples between different species. A *P* value of ≤0.05 was considered significant for all analyses. Statistical analyses were performed using JMP13 (SAS Institute, Cary, NC, USA).

Sequences were also binned into operational taxonomic units (OTUs) at a 3% dissimilarity level using the average neighbour method. Subsampling was performed to normalize sequence number for subsequent analyses, consisting of random selection of a number of sequences from each sample that corresponded to the smallest number of sequences from an individual sample. Dendrograms were developed through production of newick-formatted tree files in mothur, with visualization using FigTree v1.4.2 (Institute of Evolutionary Biology, University of Edinburgh, Edinburgh, UK). Trees were developed based on the traditional Jaccard index (a measure of community membership) and the Yue & Clayton measure of dissimilarity (a measure of community structure that evaluates membership and relative abundances). Unweighted unifrac was used to evaluate differences between groups, based on those dendrograms. Principal coordinate analysis (PCoA) was performed to visualize differences in community membership and structure. Differentially abundant OTUs were identified using linear discriminate analysis effect size (LEfSe). [19] Samples were also evaluated using the Dirichlet multinomial mixtures method for probabilistic modeling [20] to determine whether the samples could be assigned to more than different metacommunities (enterotypes).

## Results

### Study population

Samples were collected from 92 bats; 29 (32%) *Rhinolophus hipposideros* (lesser horseshoe bat), 19 (21%) *Myotis emarginatus* (Geoffroy’s bat), 11 (12%) each of *M*. *myotis* (greater mouse-eared bat) and *M*. *blythii* (lesser mouse-eared bat), five (5.4%) *Myotis daubentonii* (Daubenton’s bat), four (4.3%) *Barbastella barbastellus* (Barbastelle bat), three (3.3%) each of *Miniopterus schreibersii* (Schreibers’ bent-wing bat), *Myotis bechsteinii* (Bechstein’s bat), and *Myotis nattererii* (Natterer’s bat), two (2.2%) *Pipistrellus pipistrellus* (common pipistrelle) and one (1.1%) each of *Plecostus auritus* (brown long-eared bat) and *Rhinolophus ferrumequinum* (greater horseshoe bat). Sixty seven (73%) were males, 21 (23%) were females and sex was undetermined for 4 (4.3%). Sixty-two (67%) were adults, eight (8.7%) were subadults and 18 (20%) were juveniles. Juveniles and adults were combined for further analysis. Age was undetermined for four (4.4%). There was disproportionate representation of ages and sexes between species, with adults representing 34% (10/29) of *R*. *hipposideros*, 45% (5/11) of *M*. *myotis*, 95% (18/19) in *M*. *emarginatus* and 91% (10/11) of *M*. *blythii* (*P*<0.001), necessitating subgroup analysis.

### Sequence metrics

A total of 6,909,990 sequences passed all quality control filters, ranging from 14,455 to 152,372 per sample (median 73,937, mean 75,109). The entire database was used for comparison of relative abundances, while samples were normalized by subsampling of 14,455 sequences per sample for calculation of alpha and beta diversity. Median Good’s coverage was 0.978 (median absolute deviation 0.00979).

### Taxonomy

Twenty eight different phyla were identified, but only four (Proteobacteria, Firmicutes, Bacteroidetes and Actinobacteria) accounted for 1% or more sequences overall. Many phyla were quite rare, as the 15 phyla with the lowest relative abundances accounted for only 0.07% of sequences. Five genera from the phylum Proteobacteria (*Pseudomonas*, *Staphylococcus*, *Carnobacterium*, an unclassified Enterobacteriaceae, *Acinetobacter*) accounted for 50% of sequences. The relative abundance of Proteobacteria was particularly high in *M*. *blythii* and *R*. *hipposideros*.

**Fig 2:**
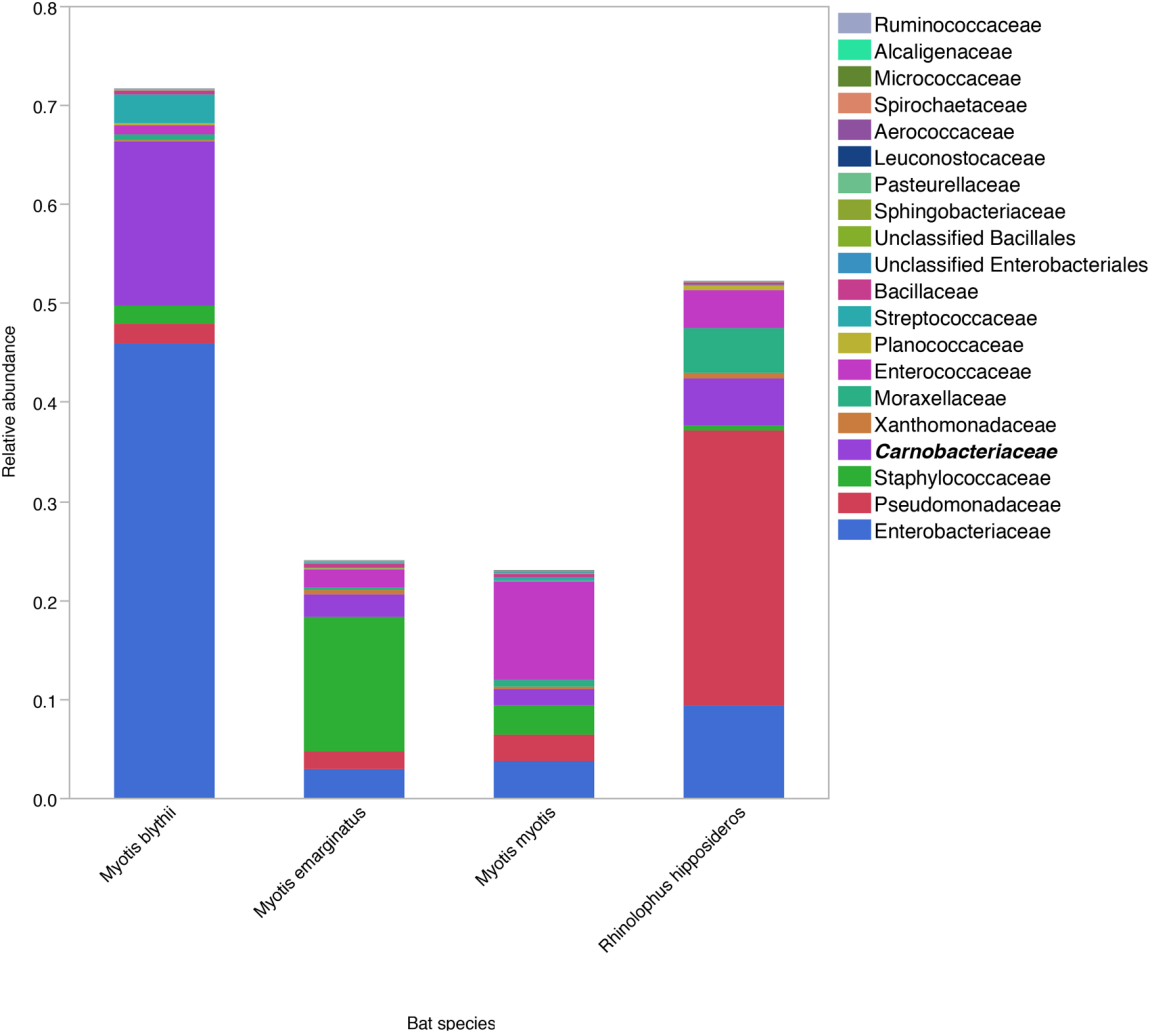
Comparison of family level data. Median relative abundances of predominant families in the fecal microbiota of *Myotis blythii* (n = 11), *M*. *emarginatus* (n = 29), *M*. *myotis* (n = 11) and *Rhinolophus hipposideros* (n = 29).

**Fig 3:**
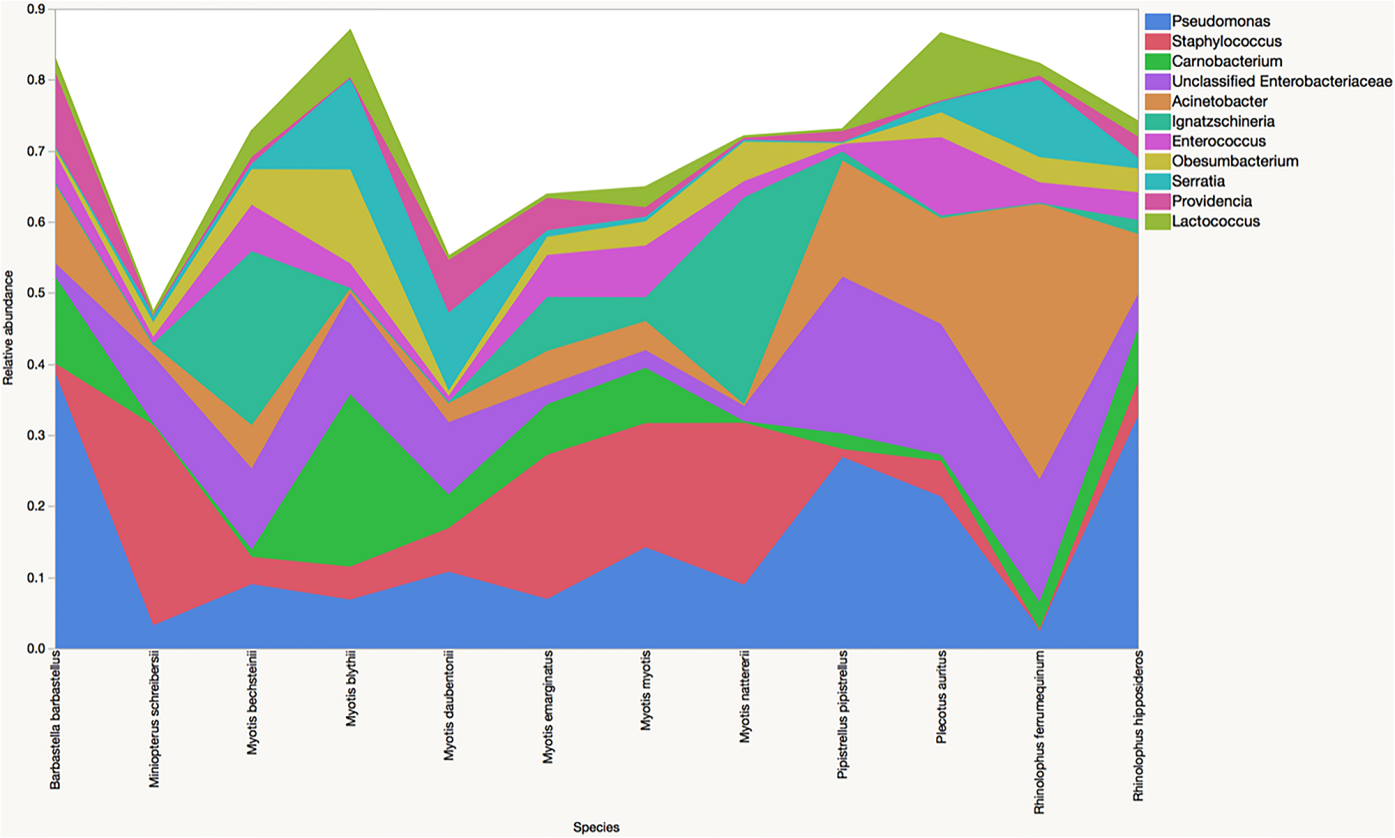
Comparison of genus data. Median relative abundances of predominant genera in the fecal microbiota of *Barbastella barbastellus* (n = 4), *Miniopterus schreibersii* (n = 3), *Myotis bechsteinii* (n = 3), *Myotis blythii* (n = 11), *M*. *daubentonii* (n = 5), *M*. *emarginatus* (n = 29), *M*. *myotis* (n = 11), *M*. *natterii* (n = 3), *Pipistrellus pipistrellus* (n = 2), *Plecotus auritus* (n = 1) and *Rhinolophus ferrumequinum* (n = 1) and *R*. *hipposideros* (n = 29).

Relative abundances of the ten most common phyla in the four major bat species (all ages combined) are presented in Table 2. There were no statistically significant differences in the relative abundances of any phyla between bat species, perhaps in part due to the the marked inter-individual variation. Various differences were noted at lower taxonomic levels. Predominant families are displayed in Fig 2. There were significant differences in Enterobacteriaceae (*P* = 0.007, most abundant in *M*. *blythii*), Pseudomonadaceae (*P* = 0.007, most abundant in *R*. *hipposideros*) and Chlamydiaceae (*P* = 0.04, most abundant in *M*. *myotis*). Predominant genera are displayed in Fig 3. Significant differences were identified for *Pseudomonas* (*P* = 0.006), an unclassified Enterobacteriaceae (*P* = 0.002) and *Serratia* (*P* = 0.002).

**Table 2:** Median relative abundances, with absolute median deviation, of the ten predominant phyla from the fecal microbiota of four bat species.

Phylum	*Myotis blythii* (n = 11)	*M*. *emarginatus* (n = 19)	*M*. *myotis* (n = 11)	*Rhinolophus hipposideros* (n = 29)
Proteobacteria	0.47 (0.321)	0.13 (0.629)	0.13 (0.299)	0.54 (0.105)
Firmicutes	0.14 (0.335)	0.21 (0.624)	0.15 (0.336)	0.13 (0.109)
Bacteroidetes	0.003 (0.000122)	0.005 (0.00168)	0.026 (0.00254)	0.005 (0.000463)
Actinobacteria	0.0011 (0.0002)	0.0014 (0.00368)	0.0031 (0.00281)	0.0065 (0.00621)
Spirochaetes	0.00022 (0.000126)	0.00054 (0.000548)	0.00020 (0.000146)	0.00015 (0.000136)
Chlamydiae	0.00015 (0.001)	0.0001 (0.000446)	0.00036 (0.000345)	0.00003 (0.000031)
Deinococcus-Thermus	0.0011 (0.000233)	0.0012 (0.00316)	0.0007 (0.000185)	0.0016 (0.00122)
Verrucomicrobia	0.00020 (0.000133)	0.00039 (0.00117)	0.00033 (0.000298)	0.00020 (0.000181)
Chloroflexi	0.00002 (0.000016)	0.00009 (0.000335)	0.00013 (0.000115)	0.00024 (0.000242)
TM7	0.00004 (0.000043)	0.00013 (0.000653)	0.00012 (0.000091)	0.00007 (0.000069)

**Fig 4:**
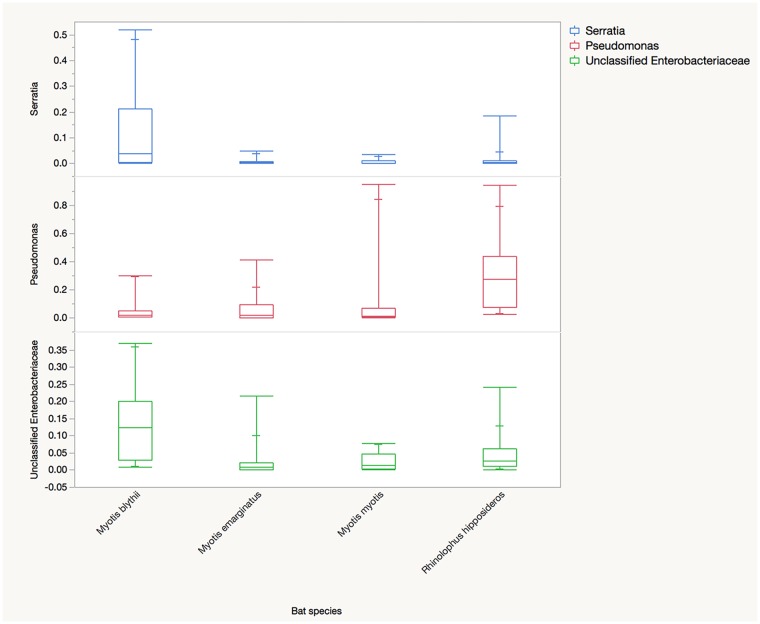
Comparison of predominate genera in adult bats. Comparison of the median relative abundances of three predominant genera in the fecal microbiota of adult *Myotis blythii* (n = 10), *M*. *emarginatus* (n = 18), *M*. *myotis* (n = 5) and *Rhinolophus hipposideros* (n = 10).

Because of the differences in age distribution between bat species, sub-analyses were performed. When only adults are considered, there were no differences between phyla. There were few differences at lower taxonomic levels (data not presented). At the genus level, the relative abundances of *Pseudomonas* (*P* = 0.028), an unclassified Enterobacteriaceae (*P* = 0.027) and *Serratia* (*P* = 0.028)(Fig 4) were significantly different. The most common genera for the main bat species are reported in Table 3.

**Table 3:** Genera with the highest median relative abundances (plus absolute median deviation) in the fecal microbiota of adult bats of four bat species.

*Myotis blythii* (n = 10)	*M*. *emarginatus* (n = 18)	*M*. *myotis* (n = 5)	*R*. *hipposideros* (n = 10)
Unclassified Enterobacteriaceae (0.31, 0.0758)	*Staphylococcus* (0.23, 0.107)	*Pseudomonas* (0.18, 0.0145)	*Pseudomonas* (0.32, 0.175)
*Carnobacterium* (0.27, 0.163)	*Carnobacterium* (0.087, 0.0138)	*Staphylococcus* (0.13, 0.0282)	*Acinetobacter* (0.100.0216)
*Lactococcus* (0.066, 0.0282)	*Pseudomonas* (0.065, 0.0169)	*Ignatzschineria* (0.098, 0.000735)	*Carnobacterium* (0.091, 0.040)
*Staphylococcus* (0.050, 0.0137)	*Enterococcus* (0.065, 0.00119)	*Weissella* (0.089, 0.00021)	*Staphylococcus* (0.089, 0.0031)
*Pseudomonas* (0.049, 0.0145)	*Ignatzschineria* (0.062, 0.00269)	*Acinetobacter* (0.082, 0.00639)	Unclassified Enterobacteriaceae (0.072, 0.0193)
*Obesumbacterium* (0.041, 0.00462)	Unclassified Gammaproteobacteria (0.045, 0.0125)	*Wohlfahrtiimonas* (0.043, 0.00187)	*Yersinia* (0.064, 0.0024)
*Enterococcus* (0.035, 0.0056)	Unclassified Enterobacteriaceae (0.042, 0.0719)	*Vagococcus* (0.039, 0.00763)	*Enterococcus* (0.043, 0.0176)
Unclassified Planococcaceae (0.031, 0.0011)	Unclassified Planococcaceae (0.038, 0.00115)	*Psychrobacter* (0.037, 0.00033)	*Obesumbacterium* (0.036, 0.00639)
*Serratia* (0.031, 0.0393)	*Providencia* (0.032, 0000281)	*Paenalcaligenes* (0.029, 0.004)	*Providencia* (0.029, 0.0057)
*Sporosarcina* (0.015, 0.000059)	*Obesumbacterium* (0.022, 0.00105)	*Enterococcus* (0.025, 0.0345)	*Serratia* (0.015, 0.00303)

Comparison of adult vs younger *R*. *hipposideros* yielded no significant differences at any taxonomic level. Comparison of ages of other bat species was not performed because of the small sample sizes.

The presence of selected genera containing potentially zoonotic species was also assessed based on previous reports in bats. *Salmonella* was identified in 18 (20%, median 0%, range 0–0.0174%) samples, *Campylobacter* in 41 (44%, median 0%, range 0–0.131%) and *Clostridium* in 90 (98%, median 0.0068%, range 0–3.99%). *Bartonella* was identified in 39 (42%, median 0%, range 0–3.80%) samples, *Rickettsia* in 16 (17%, median 0%, range 0–0.086%) and *Coxiella* in two (2.2%, median 0%, range 0–0.0156%). There was no association between the relative abundance of any of these taxa and age, sex, bat species or bat genus (all *P*>0.10). However, there was an association between the presence of *Rickettsia* and bat genus, with a significantly higher prevalence in *Rhinolophus* (28%) compared to *Myotis* (8.2%). There was no such association for other genera.

### Alpha diversity

There were significant differences in bacterial richness between species in both adults and juveniles/subadults, but no differences in diversity or evenness (Table 4). Alpha diversity was also compared between adult and juvenile/subadult *R*. *hipposideros*, and no significant differences were identified (all *P*>0.15). There was no impact of sex on any alpha diversity index either overall or with *R*. *hipposideros* (all *P*>0.28).

**Table 4:** Median alpha diversity values for fecal microbiota of the four main bat species.

	Index	*Myotis blythii* (n = 10/1)*	*M*. *emarginatus* (n = 18/1)	*M*. *myotis* (n = 5/6)	*Rhinolophus hipposideros* (n = 10/19)
Combined	Observed richness	317^a^	402^a,b^	458^a,b^	700^b^
	Estimated richness	811^a^	933^a^	918^a,b^	2279^b^
	Evenness	0.354^a^	0.379^a^	0.422^a,b^	0.459^b^
	Diversity	4.32^a^	4.69^a^	5.42^a,b^	8.64^b^
Adults	Observed richness	304^a^	303^a,b^	458^a,b^	675^b^
	Estimated richness	762^a^	927^a,b^	918^a,b^	1806^b^
	Diversity	3.95	4.47	5.42	7.51
	Evenness	0.35	0.37	0.32	0.40
Juvenile/subadult	Observed richness	NT	NT	343	606
	Estimated richness	NT	NT	1142	2359
	Diversity	NT	NT	5.3	9.6
	Evenness	NT	NT	0.37	0.50

Within rows, different superscripts indicate *P*<0.05.

NT: Not tested because of small sample size.

* numbers represent adults/non-adults

### Beta diversity

**Fig 5:**
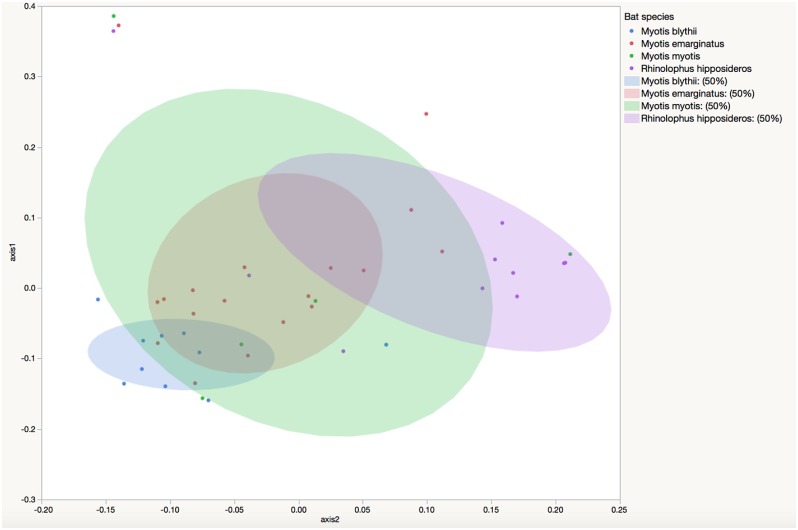
Community membership of predominant bat species. Principle coordinate analysis depicting the community membership of the fecal bacterial microbiota of *Myotis blythii* (green, n = 11), *M*. *emarginatus* (red, n = 29), *M*. *myotis* (blue, n = 11) and *Rhinolophus hipposideros* (purple = 29), with 50% ellipsoid coverage.

When only adults are considered, there were significant differences in community membership, as determined by unifrac, between *M*. *blythii* and *M*. *emarginatus* (*P* = 0.011), and *M*. *blythii* and *R*. *hipposideros* (*P* = 0.004)(Fig 5). There were also significant differences in community structure between *M*. *blythii* and *M*. *emarginatus* (*P* = 0.025), and *M*. *blythii* and *R*. *hipposideros* (*P* = 0.026).

Comparison between adults and younger bats was also performed using *R*. *hipposideros* alone, and no differences in community membership (*P* = 0.57) and structure (*P* = 0.85) were identified.

### LEfSe

When adults of the four main species were compared, 14 OTUs were identified as differentially abundant using LEfSe (Table 5). Only one difference was identified when comparing *R*. *hipposideros* adults and juvenile/subadults, with *Klebsiella* over-represented in the younger bats (LDA score 2.9).

**Table 5:** Linear discriminant analysis effect size (LEfSe) results.

*Myotis blythii*	*M*. *emarginatus*	*M*. *myotis*
*Lactococcus* (Firmicutes)	*Providencia* (Proteobacteria)	Unclassified Lactobacillales (Firmicutes)
*Serratia* (Proteobacteria)	*Aerococcus* (Firmicutes)	*Vagococcus* (Firmicutes)
Unclassified Enterobacteriaceae (2 OTUs, Proteobacteria)	*Staphylococcus* (Firmicutes)	*Weissella* (Firmicutes)
*Pantoea* (Proteobacteria)	Unclassified Gammaproteobacteria (Proteobacteria)	
	*Persicirhabdus* (Verrucomicrobia)	

LEfSe analysis identified differentially abundant operational taxon units (phylum in brackets) in the fecal bacterial microbiota of adults of four major bat species; *Myotis blythii* (n = 10), *M*. *emarginatis* (n = 18), *M*. *myotis* (n = 5) and *R*. *hipposideros* (n = 10)

### Core microbiota

No OTUs were present in all samples at a minimum relative abundance of 1%. One OTU (*Carnobacterium*) was present in 76% (70/92) of samples at that minimum relative abundance, while *Staphylococcus* was present in 68% (63/92) and an unclassified Enterobacteriaceae was present in 54% (50/92).

## Discussion

This study provides insight into the fecal microbiota of a selection of bat species from central Europe. At a high level, the dominance of the phyla Proteobacteria, Firmicutes, Bacteroidetes and Actinobacteria is consistent with the fecal microbiota of other mammals [21–26]. However, within that group of common phyla, Proteobacteria typically accounts for a smaller percentage of the microbiota than was noted here. [27–34] The median relative abundances of this phylum in *M*. *blythii* (47%) and *R*. *hipposideros* (54%) were particularly high. A cloning-based study of eight frugivorous and three insectivorous bats from India also reported predominance of Proteobacteria, [9] while members of the Enterobacteriaceae family (phylum Proteobacteria) were most commonly identified in a small culture-based study of the intestinal microbiota of the short-nosed fruit bat (*Cynopterus brachyotis*). [35] In a recent next-generation sequencing of six fecal samples from four different insectivorous species, Proteobacteria was the second most common phylum, but still accounted for 32% of sequences. [36] However, in mammals increases in the relative abundance of Proteobacteria have been widely associated with dysbiosis, being found in various disease states. [25, 37, 38] While detailed clinical history is not available, overt abnormalities were not identified in any of these bats, and the authors are not aware of any confirmed or anecdotal issues with abnormal bat morbidity or mortality in the bat population during the time of this study. It seems that the abundance of Proteobacteria in insectivorous bats could be more likely related to their phylogeny and their association with caves (environment) than their diet [36, 39]. Proteobacteria, along with Acidobacteria, and Actinobacteria, are the dominant taxa on cave walls worldwide. [40–42]

Various genera accounted for the Proteobacteria sequences, with *Pseudomonas* being amongst the most common. While unusual compared to other mammals, this is consistent with a recent digestion gradient gel electrophoresis (DGGE)-based study suggested that *Pseudomonas* was one of the most common genera in the fecal microbiota of hibernating *R*. *euryale*. [43] This genus is most often implicated as an opportunistic pathogen; however, some *Pseudomonas* from the skin microbiota of bats have been shown to be inhibitory *in vitro* against *Pseudogymnoascus destructans*, [11] the cause of the devastating white-nose syndrome. Associations between the bat’s microbiota and the pathogenesis of this syndrome warrant further investigation. Members of the class Clostridia, a predominant member of the phylum Firmicutes, were relatively uncommon, particularly compared to studies of other mammals. This group, especially members of the Clostridiales order, has often been associated with gastrointestinal health in other species. [25, 44–46] A major challenge with microbiota studies is putting the abundant and complex data into broader applied contexts. Whether the differences noted here represent potential health problems is unclear but bears consideration.

Bats are known to harbour a wide range of potential bacterial zoonotic pathogens. A previous microbiota study reported identification of genera associated with zoonotic species, namely *Coxiella*, *Bartonella* and *Rickettsia*. [36] Here, *Bartonella*, *Rickettsia* and *Coxiella* sequences were identified. Interpretation of these results is a challenge. Broad range next generation sequencing approaches are not optimal for identification of low abundance members of a community and do not differentiate individual species. The public health relevance is also unclear, because of the lack of speciation within those genera and limited likelihood of exposure of some pathogens, particularly arthropod-borne pathogens like *Bartonella* and *Rickettsia*. Enteropathogenic bacteria may be of greater relevance, but the lack of ability to speciate common and diverse genera such as *Clostridium* and *Escherichia* limits any conclusions that can be made about potential zoonotic risks. Thus, care should be taken when making inferences about shedding of zoonotic pathogens. Targeted efforts, such as through culture or species-specific PCR, are needed to properly assess the prevalence of individual species.

Richness is an alpha diversity index that indicates the number of different members (e.g. species) in a community. Significant differences in richness were identified, with richness being higher in both adult and subadult *R*. *hipposideros* compared to *M*. *blythii*. Differences in beta-diversity were also identified, with most differences involving *M*. *blythii*. Reasons for these differences are not readily apparent but may relate to exposure to different bacteria during feeding, from insect prey or the environment. Limited information is available about the environment or diet of these bat species at the time and location of sampling. The potential impact of prey on the microbiota is highlighted by the commonness of insect associated genera such as *Ignatzschineria*, *Paenalcaligenes* and *Wohlfahrtiimonas*.

Only a modest impact of age was identified in this study. While the impact of age on the microbiota has been well established in many species, the microbiota tends to stabilize relatively early in development, prior to adulthood. [21, 47] Juvenile bats, while still growing, may have already largely developed an ‘adult’ microbiota. Presumably, a greater impact of age would have been noted if younger bats were sampled. However, access to different ages was limited to the ages of bats that were caught during sampling. The number of younger bats was limited so care must be taken with any conclusion of the impact of age, but these data suggest that differences between juvenile and adult bats are limited, at least for *R*. *hipposideros*.

It is unclear how well these results can be extrapolated to other bat species and bats from other regions. There are over 1200 different species of bats, with marked differences in habitat, diet, range and size. There are potential impacts of geography, diet, environmental exposures and likely a range of other variables on the microbiota. Therefore, further study of bats in different regions would be useful to determine how conserved the microbiota is between species and regions. As part of that, identification of whether there is a true ‘core’ microbiota would be interesting. Core members, taxa that are present in most or all bats, or at least bats with similar habitats or diets (e.g. insectivores) would likely represent bacteria that have evolved closely with bats and that may play more important physiological or nutritional roles. In this study, *Carnobacterium* was the most widely distributed of the common (≥ 1% relative abundance) OTUs. This Firmicutes member (class Bacilli, Order Lactobacillales) has been studied most as a cause of packaged meat spoilage, [48] but has been found in a range of sources and sites, including dust, shrimp, the nasopharyngeal microbiota of cattle, donkey milk and feces of various species. [49–53]

Bats harbour a diverse and complex bacterial microbiota that can vary between species and age groups. There appear to be fundamental differences in the composition of the fecal microbiota when compared to other mammalian species, and further study of variation between and within bat species, as well as the impact of the microbiota on health, is indicated.
